# C-753-01. Identifying Risk Factors for Inpatient Falls in Burn Patients: A Retrospective Cohort Analysis

**DOI:** 10.1093/jbcr/irag033.022

**Published:** 2026-04-06

**Authors:** Ana G Rivera Juarez, Charles D Voigt, Sadaf Akbari, Colette Galet, Alexander Kurjatko

**Affiliations:** University of Iowa Burn Center, Iowa City, IA; Lincoln Surgical Group, Lincoln, NE; University of Iowa Burn Center, Iowa City, IA; University of Iowa, Iowa City, IA; University of Iowa, Iowa City, IA

## Abstract

**Introduction:**

Inpatient falls are a significant source of morbidity and mortality, particularly in high-risk populations. They are associated with prolonged hospital stays, increased complications, and substantial economic burden. Burn patients may face unique risks due to impaired mobility, systemic stress responses, and restrictions from dressings. Yet, the risk factors and outcomes associated with inpatient falls in burn units remain underexplored. This study evaluates the impact of inpatient falls on adult burn outcomes and identifies predictors of fall risk to guide prevention strategies.

**Methods:**

This retrospective cohort study included adult patients admitted for burn injury between July 1, 2015, and June 30, 2024. Patients who experienced falls during hospitalization were identified through an internal auditing system (Riskonnect) and matched 1:2 to non-fall controls based on age, sex, total burn surface area (TBSA), and inhalation injury. Data collected included demographics, comorbidities, medication use, Fall Risk Assessment Score (FRAS), physical therapy (PT) and occupational therapy (OT) assessments and fall safety measures. Univariate analyses, receiver operating characteristic (ROC) curve, and stepwise multivariate logistic regression were used to assess associations with fall risk. Statistical significance was defined as *p*<.05.

**Results:**

A total of 132 patients were included (45 fallers, 87 non-fallers). Fallers were more likely to use antihypertensives (62.2% vs 39.1%, *p*=.016) and antiarrhythmics (75.6% vs 52.9%, *p*=.014) and to require greater mobility assistance at admission (Basic mobility score 16 [11.3–20] vs 20 [14–24], *p*=.002). More than half (57.8%) experienced a fall prior to their first surgery, with a median time to fall of 9 days. FRAS showed moderate predictive value (AUC = 0.653, *p*=.009), with a cutoff of 15 yielding 50% sensitivity and 72–74% specificity. OT assessments reflected lower activities of daily living (ADL) independence in fallers (Daily activity total score 16 [12–19] vs 19 [14–22], *p*=.004). On multivariable analysis, burn surgery requirement (Odds Ratio (OR) = 3.35 [1.12–10.1], *p*=.031) and FRAS (OR = 1.12 [1.04–1.20], *p*=.003) were independently associated with fall risk.

**Conclusions:**

Falls in the burn unit are preceded by identifiable functional deficits and medication patterns. FRAS and early rehabilitation assessments provide valuable risk stratification tools and opportunities for timely intervention.

**Applicability of Research to Practice:**

These findings highlight the importance of functional risk assessment, interdisciplinary care coordination, and proactive fall prevention protocols tailored to the burn population.

**Funding for the Study:**

N/A.

